# The Surviving Sepsis Campaign: research priorities for the administration, epidemiology, scoring and identification of sepsis

**DOI:** 10.1186/s40635-021-00400-z

**Published:** 2021-07-02

**Authors:** Mark E. Nunnally, Ricard Ferrer, Greg S. Martin, Ignacio Martin-Loeches, Flavia R. Machado, Daniel De Backer, Craig M. Coopersmith, Clifford S. Deutschman, For the Surviving Sepsis Campaign Reasearch Committee, Massimo Antonelli, Judith Hellman, Sameer Jog, Jozef Kesecioglu, Ishaq Lat, Mitchell M. Levy

**Affiliations:** 1 New York University School of Medicine, New York, NY USA; 2 Intensive Care Department, Vall d’Hebron University Hospital, Barcelona, Spain; 3 Shock, Organ Dysfunction and Resuscitation (SODIR) Research Group, Vall d’Hebron Institut de Recerca, Barcelona, Spain; 4 Division of Pulmonary, Allergy, Critical Care and Sleep Medicine, Department of Medicine, Grady Memorial Hospital and Emory Critical Care Center, Emory University, Atlanta, GA USA; 5 Multidisciplinary Intensive Care Research Organization (MICRO), Department of Intensive Care Medicine, St. James’s University Hospital, Trinity Centre for Health Sciences, Dublin, Ireland; 6 Hospital Clinic, IDIBAPS, Universidad de Barcelona, CIBERes, Barcelona, Spain; 7 Universidade Federal de São Paulo, Sao Paulo, Brazil; 8 Chirec Hospitals, Université Libre de Bruxelles, Brussels, Belgium; 9 Department of Surgery and Emory Critical Care Center, Emory University, Atlanta, GA USA; 10 Department of Pediatrics, Cohen Children’s Medical Center, Northwell Health, New Hyde Park, NY USA; 11 The Feinstein Institute for Medical Research/ Elmezzi Graduate School of Molecular Medicine, Manhasset, NY USA

**Keywords:** Epidemiology, Organ dysfunction, Outcomes, Priorities, Research, Screening, Sepsis, Septic shock, Surviving Sepsis Campaign

## Abstract

**Objective:**

To identify priorities for administrative, epidemiologic and diagnostic research in sepsis.

**Design:**

As a follow-up to a previous consensus statement about sepsis research, members of the Surviving Sepsis Campaign Research Committee, representing the European Society of Intensive Care Medicine and the Society of Critical Care Medicine addressed six questions regarding care delivery, epidemiology, organ dysfunction, screening, identification of septic shock, and information that can predict outcomes in sepsis.

**Methods:**

Six questions from the Scoring/Identification and Administration sections of the original Research Priorities publication were explored in greater detail to better examine the knowledge gaps and rationales for questions that were previously identified through a consensus process.

**Results:**

The document provides a framework for priorities in research to address the following questions: (1) What is the optimal model of delivering sepsis care?; (2) What is the epidemiology of sepsis susceptibility and response to treatment?; (3) What information identifies organ dysfunction?; (4) How can we screen for sepsis in various settings?; (5) How do we identify septic shock?; and (6) What in-hospital clinical information is associated with important outcomes in patients with sepsis?

**Conclusions:**

There is substantial knowledge of sepsis epidemiology and ways to identify and treat sepsis patients, but many gaps remain. Areas of uncertainty identified in this manuscript can help prioritize initiatives to improve an understanding of individual patient and demographic heterogeneity with sepsis and septic shock, biomarkers and accurate patient identification, organ dysfunction, and ways to improve sepsis care.

## Introduction

As a consequence of the findings from the methodologies used by the Sepsis-3 Task Force and the 2016 Surviving Sepsis Campaign Guidelines [1], a Research Committee was charged with describing the state of knowledge and setting research priorities for sepsis by the Society of Critical Care Medicine (SCCM) and The European Society of Intensive Care Medicine (ESICM). The joint publication “Surviving Sepsis Campaign: research priorities for sepsis and septic shock” described the output of Delphi process deliberations [2]. The initial manuscript was meant to be followed by subsequent articles expounding on key areas of interest. Among these were administrative concerns, epidemiology and the scoring and identification of sepsis.

## Methods

As part of a modified Delphi consensus process, members of the Research Committee deliberated research questions to be prioritized. Each member initially submitted five research questions on whatever sepsis topic they thought most important. Through a voting prioritization process [2], these questions were reduced and subsequently categorized. An initial 88 questions were reduced to 22 final clinical questions. Subcommittees were tasked with elaborating on their initial contributions, and are previously published [3, 4]. Another document specifically addresses research priorities for Coronavirus Disease 2019 [5]. For this manuscript, subcommittees were tasked with generating expanded reviews of remaining questions. Two questions addressed administration and epidemiology, and four questions addressed scoring and identification. For efficiencies, the two subcommittees were combined and charged with producing a single manuscript.

Each question was assigned to a subcommittee member for further elaboration. Each elaborating author was asked to provide further background to the research question by addressing what is known, what is not known/gaps in knowledge, and provide future directions for research priorities. These contributions were circulated among the group and harmonized into a single document. Editing was managed by all members and further input given by the committee co-chairs. We present the results of this process. The findings presented pertain to the adult sepsis guidelines, but many be relevant to pediatric sepsis too.

## Results

### What is the optimal model of delivering sepsis care?

The Surviving Sepsis Campaign supports a structured approach to sepsis care that is based on process measures and care bundles. Such structure might raise awareness about the problem and provide a consensus approach to its diagnosis and treatment, emphasizing measures that are supported by existing science. To realize the guidelines’ full potential, science supporting the recommendations must clarify not only the benefits and risks of individual interventions, but how these interventions, in combination and applied in a complex clinical environment, improve the process of care to realize better outcomes.

#### What is known

Sepsis is a time-dependent condition in which hourly delays in diagnosis or in the initiation of effective treatments are associated with adverse outcomes and higher costs [6–10]. Unfortunately, the early identification of sepsis among patients is challenging [11, 12]. Sepsis improvement programs exist to mitigate difficulties in diagnosis, aid the implementation of evidence and improve sepsis care and clinical outcomes.

Education about best practices in sepsis has benefitted the clinical approach to patient care [13]. Irrespective of the setting of presentation, sepsis performance improvement programs lead to improved outcomes. Data suggest that programs work by enriching clinical judgment [14–16]. Importantly, sepsis education programs improved patient survival [17]. Programs focused on early detection, timely administration of antibiotics, control of infection, appropriate initial resuscitation, bundle compliance, critical task completion, and achievement of important endpoints [18–23]. Comprehensive approaches to provide sepsis education to clinicians of every specialty, especially nurses, advanced practice providers and physicians, are among the demonstrably effective strategies [17]. Of particular value were approaches such as e-learning sepsis modules, reference pocket guides, and posters containing sepsis bundles and antibiotic algorithms.

Best practices and goals are sometimes controversial, and incentivizing timely implementation can introduce trade-offs. For example, improvements associated with the early provision of antibiotics may increase the risk of administering the wrong drug [24]. The setting also matters. Prehospital antibiotics for suspected sepsis cases were not clearly associated with better outcomes, although they have not been studied for more severe sepsis presentations [25].

Mandatory quality improvement programs improved sepsis bundle compliance and enhanced sepsis care [9, 26–29]. However, these models of care delivery raise concerns about the relative importance of clinical judgment versus standardized care. Studies assessing physician adherence to inflexible quality metrics suggested that flexibility should be allowed [30]. Data indicate that performance incentive policies should focus on learning (e.g., sharing of best practices, improving the workflow of care processes) [31]. The evidence suggests that education-based quality improvement approaches might enhance the willingness of healthcare professionals to adopt sepsis performance improvement programs.

Locally directed protocols that used standard criteria and outcomes enhanced multi-professional involvement and coordination [13] and improved efficiencies and outcomes, establishing unified management criteria [32, 33]. In contrast to assigning responsibility to individual groups of clinicians, team-based strategies facilitated process management across specialties, ensuring continuity of care, adequate coordination, and staff inclusion to achieve measurable endpoints [34]. Time to intervention is often emphasized as a metric for improvement [35], and early antibiotics correlated with improved survival in septic shock [36]. Sepsis bundles, based on educational programs, were created to standardize care, avoid missed treatment opportunities, and enhance awareness [37]. Implementation of bundles correlated with improved outcomes [38].

Ineffective communication and coordination of care may lead to adverse consequences and patient harm. Medical lapses, misadventures, delays, conflicting information, repeated tests, polypharmacy, higher costs, increased workload, and reduced confidence in decisions can result from suboptimal professional interactions, either through uncoordinated activity or poor shared situational awareness [39–42]. Structured written communications, enhancing work flows and feedback have been proposed as methods to improve the process of communication in healthcare [34].

*Key points:*

- Timely application of appropriate therapies is associated with improved outcomes in sepsis care.
- Improvement programs can have unwanted effects, such as the delivery of unnecessary therapies when the diagnosis is uncertain.
- Effectiveness of quality improvement programs correlates with enhancing clinical judgment, speeding delivery of care, establishing clear responsibilities, and facilitating coordination between clinicians.

#### What is not known/gaps in knowledge

Although bundles are associated with improved outcomes, there is limited understanding about sources of effectiveness or how to test them. Key questions exist for the roles of coordination, teamwork and group cognition. An important knowledge gap concerns the roles for standardization and flexibility to account for local and patient-specific conditions. Endpoints, their measurement, and how results influence performance need further explanation.

A knowledge gap exists regarding strategies for health care modeling in different environments. Educational research is lacking to define and address gaps in understanding about best practices. Using care standardization as an endpoint requires ongoing measurement against an evolving evidence base, as standards and best practices change over time. Local factors that might drive variability are inadequately described. How knowledge gaps lead to variability in implementation of sepsis care is largely unknown.

Current detection strategies emphasize rapid identification of patients at risk of further clinical deterioration. The time-dependence of detection and interventions could be better described. Uncertainty in sepsis research investigating the timing of care delivery relates to an inconsistent approach to “time zero” for therapies. Controversies about timing trade-offs for therapies such as antibiotics persist, and are informed by limited data that illustrate either risks or benefits, but not both. Tradeoffs may vary by location and situation.

Studies of out-of-hospital antibiotic administration do not adequately address severity of illness, which might influence the benefits of timely administration. Although protocols are appealing to standardize care, they require validation, due to a lack of understanding of the factors that make them optimal [43–45]. The clinical community still faces a gap in the science of protocol application in a complex environment. Relatively little is known about pre-hospital emergency care [46]. There is ongoing need for better indicators for triage of critical care resources [47].

#### Future directions

Improvements in the administration of sepsis care will come from a stronger foundation in education, organizational and cognitive sciences. Cost-effectiveness, delivery efficiencies and unwanted trade-offs should be compared. Means of improving situational awareness, interdisciplinary collaboration and communication are objectives for investigation. Cognitive and social research exploring interventions that enhance collaboration could provide useful information for the creation of more effective quality improvement strategies. Identifying key metrics, ways to measure them and provide feedback to participants might improve program effectiveness. As more effective programs result from such investigations, another future target for systems research should be a better understanding of how to enhance the willingness of healthcare professionals to adopt sepsis performance improvement programs.

Various cognitive artifacts, objects or cues that help clinicians think, might improve group consensus and sense-making. Sepsis bundles were developed as a cognitive strategy for implementing guideline recommendations, and future efforts are needed to build a better working knowledge of how bundles work and how to improve upon them to facilitate better care. Electronic medical record data management and alerting could support the early detection and efficient and timely delivery of high-quality interventions to patients with sepsis [15, 48, 49]. Artificial intelligence/machine learning tools may facilitate the processing of large amounts of data to enhance detection and treatment systems.

## Important research questions:

- What impacts, positive and negative, occur as a consequence of standardized sepsis care, taking into consideration timing, appropriateness (e.g., missed diagnosis) and effectiveness?
- What interventions enhance coordination and communication, and how can these activities be measured?
- What devices can help clinicians manage complex decision-making in the setting of sepsis?

### What is the epidemiology of sepsis susceptibility and response to treatment?

A goal of epidemiology is to infer biological rationales from population-based observations [50]. In sepsis research, epidemiology would help advance the understanding about how population data influence sepsis susceptibility, clinical course and response to therapy. Many factors play roles, including age, comorbidities and access to health care. When identified and isolated, the effects of these independent variables on relevant outcomes can be tested. Challenges include the dominance of one independent factor over others and variability in measurement. Other concerns relate to definitions and the relationship between biologic and population data when assessing prognostic/diagnostic performance.

#### What is known

Applying different sepsis definitions leads to variable assessments of sepsis epidemiology. For example, while overlap is quite high [51], Sepsis-2 definitions [52, 53] identify some patients not identified when Sepsis-3 [54] criteria are applied, and vice-versa. Similarly, different data sources affect estimates of the incidence and mortality [55]. Cause-of death-based determinations suggest that the global incidence of and mortality from sepsis are declining [56]. These same data attribute 20% of all deaths to sepsis, and indicate that 85% occur in lower- and middle-income countries, highlighting ongoing disparities and opportunities for research [57]. In contrast, analyses using clinical data suggest that the incidence of sepsis and the number of sepsis-related deaths have remained relatively static over time. Healthcare claims-based data, which are limited to the United States, indicate variability [55, 58–60]. Post hoc analysis indicates that approximately 30% of patients with a sepsis-like syndrome have a "possible" likelihood of infection [61]. Even in high-income countries, racial and gender disparities affect the risk of sepsis and mortality. Higher rates of comorbidities in some populations confound analyses of disparities [62]. Intra-country causes of disparities include structural inequities, socio-cultural factors and differences common across high-, middle- and low-income countries [59].

Using epidemiologic data like gender, ethnicity, nationality, resource availability, age, and comorbid conditions in sepsis research is difficult, in large part because of confusion arising in the criteria used to link outcomes to independent clinical variables. For example, the effects of age or coexisting illness on outcome can overwhelm the contribution of other variables. One approach to this problem is to use available data to create sepsis “subtypes”. Clinical presentation, progression, response to treatment, and risk of mortality may be affected by variations in the response of each unique host [63–65]. Biomarker-based strategies identified heterogeneity in treatment effects in sepsis, and may improve ways to assess therapies in better-selected research populations [66–69] or improve the diagnosis of infection [70]. Although reliant on molecular and pathobiology-driven characteristics, phenotypic models could enrich classic epidemiologic data [71–75]. Four sepsis clinical phenotypes correlated with clinical outcomes and host immune-response patterns [75]. Mortality in one group, which included patients who were older and sicker, was higher than in the other three. Using computer modeling to alter the characteristics of patients included in several highly cited clinical trials resulted in outcomes that differed significantly from what was observed in the actual trial. Sepsis response signatures or Molecular Diagnosis and Risk Stratification of Sepsis (MARS) clusters are another model to identify specific clinical phenotypes [72, 73, 76].

*Key points:*

- Changing sepsis criteria lead to varying estimates of incidence and mortality.
- The prevalence of comorbidities and access to health care resources in different populations contribute to different estimates in sepsis epidemiology,
- Creation of “types” using multiple data sources enhances diagnosis and prognosis estimates.

#### What is not known/gaps in knowledge

The lack of consensus on definitions/clinical criteria and on the appropriate source of data makes quantifying the global burden of sepsis challenging [77]. Confirmation of infection is also imprecise. While a number of studies have compared different approaches to data acquisition and different diagnostic criteria, parsing the effects of covariates such as age and comorbidity from one another is difficult. Given the lack of effective markers, different studies may address different populations. Study settings and methodologies are key factors in variability. Comparing data from low-resource and high-resource areas is problematic because of differences in infecting organisms, implementation of proven treatment approaches and access to critical supplies and personnel [77]. These circumstances preclude extrapolation between settings [57]. Selecting patients for studies based on the severity of disease does not account for specific host responses and other undescribed heterogeneous variables. Comparisons between studies are difficult because the contributions of pathogen- and host-specific factors on clinical progression and outcomes are not sufficiently accounted for by most commonly collected epidemiologic data. We lack adequate models to classify and select patients to evaluate specific treatments or allocate resources. Currently there is no validated means, outside of infection-specific therapies (antibiotics, source control) to individualize treatments based on patient subtypes [78].

Until definitive classification criteria are developed, estimating epidemiologic trends and identifying appropriate endpoints will remain challenging. Classifying sepsis on the basis of clinical or molecular phenotypes/endotypes must be refined if the classifications are to identify high-risk patients or evaluate therapies [79]. In spite of decades of research, biomarker-based strategies have provided neither diagnostic specificity nor identified more precise entry criteria for clinical trials [68, 71, 79]. Absent the ability to alter a biomarker or phenotype and observe a change in a biologically relevant outcome, or development of a database so large that genetic incongruities become statistically irrelevant, biomarker- or phenotype-based approaches are of limited value. Much needs to be learned about the effects of comorbid conditions on sepsis [80–82].

#### Future directions

In order to better apply population data to sepsis research, further efforts must be made to disentangle the effects of multiple independent variables on susceptibility and treatment responses. Isolating the effects of gender, race/ethnicity, nationality, and resource availability should be an explicit goal of future epidemiologic and therapeutic investigations. Investigations should determine if separating patients into subgroups could be useful earlier in the disease, affecting prognostic prediction, use of ICU resources, or guiding treatment [47, 83]. Prognostic enrichment should be tested to improve the suitability of patients to receive risky therapies and to refine enrollment in clinical trials [84]. Biological evidence may become a component of a sepsis definition and reduce heterogeneity if they can enhance population data [83, 85]. Model derivation, validation and application to published and proposed studies should become a component of trial design and analysis. Efforts should explore accruing larger datasets to account for genetic and other variabilities among patients and fit a model that accounts for major confounders, which themselves should be better characterized.

## Important research questions:

- How can investigators disentangle complex interactions to isolate important clinical factors when investigating sepsis therapies?
- What factors identify subgroups for diagnostic or prognostic enrichment of study cohorts?
- How can datasets be managed to reduce confounding or the dominance of one factor over other important ones when trying to describe sepsis subsets?

### What information identifies organ dysfunction?

Sepsis is defined as “life-threatening organ dysfunction caused by a dysregulated host response to infection” [55]. Thus, organ dysfunction is one of the three defining characteristics of the disorder. Identification of organ dysfunction is particularly important because it helps address a key concern—the absence of “gold standard” criteria that definitively demonstrate that sepsis is present. It thus becomes necessary to use a surrogate or proxy for sepsis. In many large epidemiologic studies, patients are assumed to have sepsis if they meet the test of “outcomes validity”—that is, if they are infected and arrive at some outcome consistent with sepsis. Most commonly, this outcome is death. Other examples of outcomes validity include a substantial ICU length of stay or a need for mechanical ventilation. Unfortunately, outcomes validity is rarely useful at the bedside. The clinician caring for a patient suspected of having sepsis wants to know long before most viable outcomes have been reached. The alternative approach is “construct validity”. The proxy for sepsis under this approach is that the patient develops a state that “looks like sepsis”. Construct validity is highly subjective. The ability to use organ dysfunction, a defining characteristic of sepsis, as a construct substantially removes some subjectivity, and therefore is desirable.

At times, it is difficult to identify organ dysfunction, or to differentiate it from the other defining characteristics of sepsis, infection and a dysregulated host response. For example, organ dysfunction may be dependent on the host response, and dysregulation of the host response may in turn depend on the infecting organism. Further complicating matters is terminology; dysfunction, injury and failure are often used interchangeably. For convenience, failure may be thought of as an irreversible process, while dysfunction denotes the possibility that the process can be reversed [86–88]. However, distinguishing organ dysfunction from organ injury may be more complicated because identification of both may depend on the use of another set of proxy measures—biomarkers.

#### What is known

Biomarkers for sepsis-induced dysfunction have been validated for several organ systems [71, 89, 90]. In rare cases, clinical dysfunction can be identified without requiring some marker of injury. Decreased myocardial contractility, most often identified using echocardiography, indicates myocaridal dysfunction but measuring troponin is rarely useful. More often clinical criteria for a sepsis-induced abnormality rely on biomarkers that correlate with dysfunction and/or injury [91–94]. For example, the effects of sepsis on the liver are characterized by hyperbilirubinemia and impaired clotting studies (dysfunction of bile acid transport and protein synthesis, respectively) and elevated serum levels of transaminases, indicative of hepatocellular injury.

Objective criteria exist to denote lung and kidney dysfunction [89, 90]. However, biomarkers that indicate dysfunction alone (hypoxemia, elevated creatinine) may be nonspecific and may occur late in the course of sepsis. More sensitive markers most often reflect injury (altered pulmonary compliance, abnormal chest radiography, elevated levels of KIM-1). Use of inflammatory biomarkers in patients with the acute respiratory distress syndrome (ARDS) has identified subsets (“phenotypes”) that correlate with outcomes such as response to fluids, positive end expiratory pressure (PEEP) or certain drugs [95–99]. Biomarkers for gastrointestinal, brain, peripheral nerve or skeletal muscle dysfunction and/or injury are nonspecific. Some combinations of biomarkers related to dysfunction in different organ systems have prognostic value. These include the Sequential Organ Failure Assessment (SOFA) and Multiple Organ Dysfunction (MODS) scores [100–104].

*Key points:*

- Many markers for organ dysfunction are nonspecific.
- Combinations of markers may improve the ability to detect organ dysfunction.
- Dysfunction and injury are separate processes, but frequently confused.

#### What is not known/gaps in knowledge

In most cases, the relationship between most biomarkers and actual organ dysfunction has remained elusive. The deficit in knowledge reflects the absence of gold standards, and is also a comment on the lack of studies truly validating many biomarkers. Pathobiology of organ dysfunction is not well described. In addition, the descriptors of organ dysfunction change as sepsis progresses. The relationship between dysfunction and injury in specific organ systems is poorly characterized. When testing and applying criteria for organ dysfunction, investigators and clinicians need to account for changes that reflect infection itself versus the effects of other pathologic states (e.g., shock) or insults (e.g., nephrotoxins). Counting dysfunction by organ systems does not adequately explain the concept of sepsis-associated dysfunction, nor does it explain the interrelations between different dysfunctions.

#### Future directions

In the future, clinical criteria for sepsis should be informed by patterns that reflect a disease state with expected outcomes whose trajectory can be corrected with proper therapies. Clinicians and researchers should disentangle dysfunction caused by new-onset sepsis, by sepsis perturbations (e.g., shock) or by pre-existing sepsis. A better approach to melding indices of cell damage with those of impaired organ function is fundamental. Efforts are needed to explore the relationship between mechanisms of injury and dysfunction. For example, both microcirculation changes and mitochondrial aberrancies are invoked as causes of injury. It is likely, however, that both are induced by sepsis, and their relationship to each other is unclear. Therapeutic targets of interest include prognosis markers and correction of organ dysfunction. Investigators should look for better markers that can provide actionable information before injury has already developed, and indicate a process whose culmination is sepsis-induced organ dysfunction. A key gap in understanding the pathobiology of sepsis could be addressed by efforts to integrate measureable data qualifying function and dysfunction.

A major obstacle to research is circular logic. By defining sepsis as organ dysfunction from a dysregulated response to infection but lacking a way to establish that dysfunction is sepsis-related (because sepsis is defined by organ dysfunction in the first place), clinicians and researchers constantly contend with establishing which came first. The proposed paths we have cited offer an opportunity to solve this problem and conceive of interventions to prevent further morbidity.

Understanding the ways dysfunction can be detected will aid in identifying patients for therapies. Integrating studies that measure and combine imaging, molecules and assess function into more specific patterns could help fulfill the goal of establishing validated signals of how a dysregulated response evolves. Investigation of phenotypic variation might yield useful information about individual susceptibilities to dysfunction, or help determine patterns specific to sepsis. All investigations must contend with the reality that sepsis outcomes depend on patients’ responses, comorbid conditions and baseline status, sources of infection and appropriateness of therapies. Being able to measure function and dysfunction is a way to disentangle these variables and a route to therapeutic insights.

## Important research questions:

- What is the pathobiology of organ dysfunction?
- What is the relationship between dysfunction and injury?
- How can dysfunction from sepsis be differentiated from that caused by other coexisting pathologic states (e.g., shock, toxic drugs)?

### How can we screen for sepsis in varied settings?

While epidemiologic study helps determine sepsis susceptibility and response to therapy in populations, insights can be used to identify patients for clinical care. Better description and categorization of patients supports clinical diagnosis and effective therapies. A lack of clinical criteria that effectively screen for sepsis is a major challenge. This defect has repercussions for epidemiology, treatment, quality improvement initiatives and research efforts.

#### What is known

Absent a diagnostic gold standard, outcome proxies such as mortality or ICU admission are generally used to identify patients with sepsis. These proxies calibrate a screening model, but they can be influenced by factors such as standards of care, setting and inequities. For Sepsis-3, using a SOFA score leverages available but imperfect proxies for organ dysfunction as criteria for sepsis [102]. Improvements in sensitivity, specificity, positive and negative predictive values, availability, resource consumption and performance for individual patients and populations are important to assess new screening tools.

Elements of the systemic inflammatory response syndrome (SIRS) criteria [105] have been used in previous sepsis definitions and in many clinical screening programs. While these criteria are appealingly simple, they do not account for a key component of sepsis—organ dysfunction. Indeed, the original paper advocating for the use of SIRS criteria recognized this problem by inventing a new term—severe sepsis—to include organ dysfunction [52, 105]. Evidence demonstrating that SIRS criteria are accurate or valid indicators of sepsis is limited [9, 106].

The Sepsis-3 Task Force criteria propose the quick Sepsis-Related Organ Failure Assessment (qSOFA) score as a prompt for clinicians to look for underlying organ dysfunction and sepsis in non-ICU patients [54, 55]. Predictive validity for mortality was better than SIRS and comparable to SOFA scores in the patient population examined. Studies in high- and middle-income settings demonstrated a low sensitivity for the outcomes of mortality and the receipt of critical care interventions [107, 108], and suggested low specificity [109] as a screening tool for sepsis. These studies did not investigate qSOFA as a prompt for further clinical assessment. Other studies support the validation of the qSOFA criteria as a screen, but similarly do not study the score in the context intended by its creators [110–113].

Using the full SOFA score as a screening tool is appealing in that it incorporates the concept of organ dysfunction, albeit in a limited way. Derivation and validation studies suggest it is useful in the ICU setting and that an increase in score of 2 or more is highly predictive of hospital mortality [103]. The eSOFA score is a simplified version optimized for electronic health records. In a small cohort of patients, eSOFA’s performance was better than the Sepsis-3 SOFA criteria [114]. The score uses widely available criteria and is practical to use. eSOFA may be best suited for use in retrospective review, as it negates many of the biases inherent in administrative databases.

Clinical deterioration models and early warning scores, such as the National Early Warning Score (NEWS) or the Modified Early Warning Score (MEWS) [115, 116], may predict deterioration in inpatients and assist in ED triage, but they are operationally dependent on the sensitivity cutoff point used [117, 118]. An important consideration in implementing a warning system is tuning it to local conditions in order to balance excessive alarms with delays in diagnosis. Of note, these scores are not specific for sepsis.

In resource-limited settings, screening is mostly based on available data and cannot count on costly diagnostics or the deployment of response teams. The routine monitoring of vital signs and vigilance for infection can raise suspicion of sepsis, but vital sign abnormalities can be seen in many non-sepsis conditions [119].

Predictive analytics can currently produce robust, adaptive models to sort patients by risk for various outcomes, and models exist to predict outcomes highly correlated with sepsis [120–122]. To be effective, these models must optimize performance, make timely diagnoses, and be linked to actions that improve outcomes. The analytic systems are the afferent arm to prompt timely interventions. Like warning systems, these models must minimize unnecessary alerts that overwhelm clinicians and resources.

Rapid acquisition of actionable data is crucial. Screening too early erodes specificity. Screening too late reduces the ability to mitigate deterioration and organ dysfunction. The original qSOFA validation study considered data from 48 h before to 24 h after the onset of infection [54]. This period of time remains the best described window of information, but may change based on new ways to measure dysfunction. Table 1 lists several scoring systems.

**Table 1:** Scoring systems used for patients with sepsis and the components that contribute to them

Score	Components
Systemic inflammatory response syndrome criteria	Temperature Heart rate Respiratory rate PaCO_2_ White blood cell count % immature neutrophils
Sequential Organ Failure Assessment (SOFA) Score	PaO2/FiO2 Glasgow Coma Score Mean arterial pressure Use of dopamine, dobutamine, epinephrine or norepinephrine, and dose Bilirubin Platelet count Creatinine Urine output
Quick Sepsis-Related Organ Failure Assessment (qSOFA)	Systolic blood pressure Respiratory rate Glasgow Coma Score
National Early Warning Score (NEWS)	Respiratory rate Oxygen saturation Supplemental oxygen Temperature Systolic blood pressure Heart rate Alert, Voice, Pain Unresponsive (AVPU) Score
Modified Early Warning Score (MEWS)	Systolic blood pressure Heart rate Respiratory rate Temperature AVPU Score
Sepsis-Related Organ Failure Assessment optimized for electronic health records (eSOFA)	Vasopressor initiation Mechanical ventilation initiation Creatinine Bilirubin Platelet count Lactate

*Key points:*

- Sepsis identification is based on proxies for dysfunction that are used in combination (e.g., SOFA score).
- qSOFA was conceived as a quick tool to prompt further clinical assessment for sepsis.
- Warning systems may enhance sepsis screening, but must be tuned to optimize alarm volume and effectiveness. These factors can be setting-dependent.

#### What is not known/gaps in knowledge

The best screening tool for each setting has not been determined. SOFA was developed in cohorts receiving care in high-resource environments, mostly in the United States, and may not perform as well elsewhere. The scale includes outdated therapies and measurements that may not correlate well with true organ dysfunction. An aggregate SOFA score of 2 might represent mild abnormalities in 2 organ systems or more significant abnormalities in one. It is unclear that these are equivalent states in the setting of infection. Existing proxies for organ dysfunction are limited. Linkages to important outcomes not influenced by other external variables are lacking. There are few studies of sepsis screening that take into account the mechanics of how the screening affects work flow and how the work flow changes outcomes. Currently, there is an opportunity to better harmonize definitions, diagnostics and treatments to improve sepsis outcomes. Tradeoffs in different approaches are not characterized, in particular those involving sensitivity and false positive rates, model consistency versus generalizability, detection-focused versus action-focused constructs, and targeting early cases versus all cases.

Until better markers for organ dysfunction are identified and validated, screening for sepsis based on organ dysfunction will be limited. Suspecting infection is still a good first step in screening, but there are currently limited tools to enhance the suspicion that infection is present. Although the calculation of a SOFA score as a screening tool appears reasonable, it lacks prospective validation of its usefulness at the bedside. Deterioration models currently do not use markers of deterioration that are unique to sepsis. Predictive analytics systems have performed adequately in detecting sepsis cases retrospectively, but there is no clear demonstration that their use effectively changes relevant clinical outcomes.

#### Future directions

Better screening for sepsis would include improved screening for organ dysfunction. Selecting better proxies for organ dysfunction and, more problematically, for a dysregulated host response, and validating their predictive abilities in a variety of settings are imperatives for improving screening. These proxies would ideally be easily available, capture dysfunctions early and be specific for sepsis-induced organ dysfunction. Identification of common variables (for example, those that come from better descriptions of human physiology and the dysregulated response to infection) across a variety of settings would enhance the ability to screen and treat the disease, and to study the effects of screening. Operationally, improved descriptions of relevant outcomes, and the linkage between screening tools, therapeutic responses to the screens and outcomes would help guide clinical recommendations.

Tools using data from the electronic medical records might, using algorithms, be adapted to predict cases of sepsis. There is a need to explore how algorithms could trigger appropriate diagnostic and therapeutic measures. The relevance of these algorithms may depend on the resources available and it will be important to assess whether they hold promise in lower income settings. Finding better ways to use the data available to clinicians in a variety of settings is an important focus for future research.

## Important research questions:

- What markers identify a dysregulated host response to infection?
- How can screening tools lead to actions that improve clinical outcomes?
- How can screening be implemented in resource-limited environments?

### How do we identify septic shock?

Shock has long been viewed as an extreme form of circulatory insufficiency whose association with mortality is validated [123–125]. The entity is often classified based on etiology. In most cases, the clinical characteristic that identifies shock is hypotension. Septic shock is different; its defining characteristics involve more complex biology, which primarily reflects altered cellular function, aberrant metabolism or deranged biochemistry. Identification is based more on the recognition that normally adaptive, evolutionarily conserved mechanisms either become maladaptive or fail altogether. The rationale for defining septic shock as a unique entity appears to arise from two considerations. The first reflects the need for a syndrome analogous to other forms of circulatory insufficiency. In the articles generated by the first two sepsis consensus conferences [52, 105], septic shock was loosely defined as “sepsis with hypotension”. The second involves outcome; numerous studies indicated that mortality was higher when sepsis was accompanied by hypotension.

Characterizing shock of any etiology becomes problematic when trying to determine how low the blood pressure must be for an entity to be called “shock”. In most forms, the blood pressure is deemed to be sufficiently low to be considered shock if it is associated with a clinically apparent change in organ function, e.g., an altered mental status. Other characteristics used to identify organ dysfunction include oliguria, nausea/vomiting or shortness of breath. In sepsis, organ dysfunction often precedes the development of hypotension. By definition, it has to manifest in order to diagnose sepsis. Thus, conventional criteria for identifying shock may not apply. Indeed, it is tempting to consider “septic shock” as simply “sepsis where the sepsis-induced organ dysfunction lies in the cardiovascular system”. An additional difficulty arises when considering the underlying cause of hypotension. In most forms of shock, the primary defect is known. This knowledge identifies a rational approach to therapy. In sepsis, however, the underlying defect is unknown. Indeed, virtually all the abnormalities that lead to hypotension in other shock states are present in sepsis (Table 2)—but these abnormalities are present whether or not there is hypotension.

**Table 2:** Abnormalities leading to hypotension in various shock states, including septic shock

Shock type	Venous return	Pump function	Vascular tone	Other abnormalities	Examples
Cardiogenic	Normal or ↑	**↓↓**	Normal or ↑	Primary pump failure, may develop compensatory salt/water retention and increased vascular tone	Myocardial infarction, injury
Neurogenic	↓	**↓↓**	**↓↓**	Loss of inotropic/chronotropic input and vascular tone	High cervical transection, spinal anesthetic
Distributive	**↓↓**	Normal (may ↑ or ↓, depending on etiology and compensation)	↓	Mediators dilate a vascular tree that cannot be filled with the existing blood volume	Anaphylaxis, adrenal insufficiency, SIRS response
Septic	**↓↓**	↑ or normal, but can be **↓↓**	**↓↓**	Dysfunctions in 1. Vascular tone leading to pooling of venous blood, 2. Myocardial pumping 3. Vascular tone, contributing to hypotension	Peritonitis, pneumonia, urosepsis

Key abnormalities are indicated by bold double arrow

To revise the definition of septic shock the Sepsis-3 task force sought to reconcile these different considerations. Given that sepsis-associated mortality is affected by what organ systems are dysfunctional, considering septic shock as a unique entity might not be justified. The view of shock as a state of cellular, and not organ system, dysfunction removed the requirement for hypotension. After debate, the task force arrived at a compromise. Thus, septic shock is defined as “a subset of sepsis in which underlying circulatory and cellular/metabolic abnormalities are profound enough to substantially raise mortality”. This formulation encompasses both circulatory and cellular abnormalities while acknowledging that, at this time, the main distinction between sepsis and septic shock lies in the rate of survival.

#### What is known

In Sepsis-1, septic shock is defined as “sepsis with hypotension, despite adequate fluid resuscitation, along with the presence of perfusion abnormalities that may include but are not limited to, lactic acidosis, oliguria, or an acute alteration in mental status”[105]. Sepsis-2 defines septic shock as “a state of acute circulatory failure characterized by persistent arterial hypotension unexplained by other causes”. The key criterion is hypotension “unexplained by other causes” [53]. Importantly, these “definitions” are really clinical criteria by which septic shock in patients might be recognized. Both Sepsis-1 and Sepsis-2 define hypotension as a systolic BP < 90 mmHg; Sepsis-2 adds a mean BP < 60 mmHg. Both sets of criteria are problematic. The choice of 90 mmHg is arbitrary and neither measure provides an approach to assure that hypovolemia is absent. Lactate in sepsis may be elevated in the absence of hypotension, reflecting a sepsis-induced defect in oxidative phosphorylation (“aerobic glycolysis”) or resulting from administered or endogenous catecholamines. Mental status changes are among the earliest observed clinical criteria for sepsis and may be present in the absence of septic shock. Oliguria is often an adaptive response to compensate for circulatory inadequacy and thus is a poor surrogate for renal dysfunction.

Perhaps the most important issue, however, is that both Sepsis-1 and Sepsis-2 are products of expert opinion. The choice of the variables used as clinical criteria (e.g., SBP < 90 mmHg, lactic acidosis, oliguria, etc.) was made by task force members.

The clinical criteria for septic shock enumerated in Sepsis-3 were identified based on expert opinion, but the process was more refined. A literature search identified 44 studies reporting septic shock mortality that contributed to a list of the clinical criteria for septic shock. This list was used to inform a Delphi process among the task force members [54, 102]. The task force members chose hypotension, vasopressor therapy and hyperlactatemia as criteria for septic shock. These criteria were then applied to data from patients entered into the Surviving Sepsis Campaign (SSC) database who were designated as “having adequate fluid resuscitation, met SIRS criteria and had dysfunction in one or more organ systems”. Results informed a second round of Delphi deliberations and approval of the need for vasopressors as a proxy for hypotension after resuscitation. A combination of all three variables identified cohorts of patients whose mortality was significantly higher than cohorts identified by any single variable or any combination of two variables, and were confirmed in cohorts of patients with suspected infection derived from two electronic health record (EHR) databases (54 and 35% mortality) [126].

Since the publication of Sepsis-3, a limited number of studies examining septic shock have confirmed the findings [127]. The Sepsis-3 septic shock criteria were confirmed in a lower income population using data from a public hospital in Brazil [128] and in ICUs in Asia [129–132]. Sepsis-3 criteria have been successfully applied to children [133]. Stratification using lactate levels may improve accuracy [134]. Several studies have examined the potential contribution of criteria beyond hypotension, vasopressor use and lactate level, including a variety of biomarkers [124–133, 135–139]. In contrast to the Sepsis-3 criteria for septic shock, the difficulty in obtaining measurement of most of these putative biomarkers limits their routine use (Table 2).

In perhaps the most comprehensive study to date, data from 205,632 patients identified as having sepsis using either Sepsis-1/2 or Sepsis-3 criteria were extracted from the national critical care database in England [51]. In this report, 92% of patients met both Sepsis-1/2 and Sepsis-3 criteria for sepsis. In contrast, among 153,623 patients identified as having septic shock, only 38,896 patients (25%) met both sets of criteria. Sepsis-3 criteria identified fewer (39,262 vs. 153,257) patients with septic shock than Sepsis-1/2 criteria. Less than 1% of the patients identified by Sepsis-3 criteria did not satisfy Sepsis-1/2 criteria, suggesting better discrimination of a high-mortality cohort. ICU mortality among patients meeting Sepsis-3 criteria for septic shock was 47%, significantly higher that the 22% rate among patients meeting Sepsis-2 criteria for sepsis. Conversely, ICU mortality among patients meeting Sepsis-1/2 criteria for septic shock was 26%. Importantly, all patients meeting Sepsis-3 criteria for septic shock had to meet criteria for sepsis, and most met both Sepsis-1/2 and Sepsis-3 criteria. As therapeutic approaches are the same for sepsis and septic shock patients, better discrimination need not come at the expense of missed cases.

*Key points:*

- Criteria for septic shock were produced by a consensus process and are nonspecific.
- Sepsis-3 criteria for septic shock reflect a more refined process and perform better at discriminating a high-mortality cohort while missing few cases.

#### What we do not know/gaps in knowledge

The most readily explored open questions include validation of established criteria and evaluation of hemodynamics. Septic shock variables, alone or in combination, have not been verified prospectively. Areas for study include prospective validation of Sepsis-3 clinical criteria for septic shock, further evaluation of the criteria in resource challenged populations and environments, and investigation of the use of serum lactate levels to improve discrimination. The impact of added proxies that involve non-routine and potentially expensive tools (e.g., complex technologies, lab tests) on the validity of Sepsis-3 criteria is unknown. It is important to examine the cost–benefit ratio of any added criteria. Other clinical questions include: When, relative to fluid administration, should vasopressors be initiated? and: Is there a better “vasopressor of choice” than norepinephrine?

It remains unclear if there are tangible pathobiological difference between sepsis and septic shock. Such a dichotomy would demonstrate that septic shock is an entity distinct from sepsis and not just “sepsis with CV dysfunction”. It also would eliminate mortality from the definition. Septic shock should be more than merely a subset of sepsis where mortality is substantially increased. In the current state of knowledge, we lack a “gold standard” that can be used to evaluate clinical criteria, biomarkers and other proxies. If septic shock is indeed a unique disorder, are there biomarkers that identify it without also identifying sepsis? If so, does it require treatment that is different from what is used to treat sepsis and support the circulation? Answers to these questions would support the clinical use of the term “septic shock”.

Regarding the concept of shock, a better characterization of pathobiology could inform diagnosis and therapeutics. Is shock a macroscopic or microscopic phenomenon? Is there a spectrum—from biochemical to sub-cellular (e.g., mitochondrial) to cellular to tissue/organ system to systemic manifestations (e.g., hypotension, hyperthermia)? Are there ways to differentiate between these possibilities? In other forms of shock, the initiator is known.

Another unanswered question is whether there is a chronological progression from infection to sepsis to septic shock, or if it is possible to transition from infection directly to septic shock. This should be the case if the underlying pathobiology of sepsis and septic shock are different.

#### Future directions

New definitions or identifying criteria would be expected to disentangle “septic shock” from “sepsis with dysfunction of the cardiovascular system”. If septic shock is to be a separate entity, the diagnostic approach to it should differ from that used for sepsis absent septic shock and efforts should seek to progress towards a “gold standard” for diagnosis and study. Obtaining insight as to whether underlying factors mediate the transition from simple infection to sepsis or to septic shock and justify designating septic shock as a unique entity is fundamental. Future research would specify therapeutic targets for septic shock that are independent of those attacked when treating sepsis.

## Important research questions:

- Is septic shock a distinct entity from sepsis?
- What is the pathobiology of septic shock?
- How can the presence of shock be confirmed independent of the diagnosis of sepsis?
- When should specific interventions be initiated in the setting of shock that would not otherwise be used in sepsis without shock?

### What in-hospital clinical information is associated with important outcomes in patients with sepsis?

Treating sepsis should improve clinical outcomes, but addressing what constitutes an important outcome is a challenge. Viable construct criteria for sepsis should associate with whatever outcomes are selected. Ideally, treating some or all criteria improves these outcomes, but this relationship cannot be assumed. The presumed pathobiology of sepsis suggests that most criteria serve as markers rather than drivers of disease. These criteria should have clinically relevant outcomes validity. Whereas pursuit of a definition might improve the description of what sepsis is, clinical needs focus more on the parameters that can trigger therapies that improve outcomes.

Absent defining features, sepsis is either inferred subjectively (i. e., “I know it when I see it” [140]) or approximated by objective, imperfect proxies. Recognizing and appropriately treating a dysregulated host response to infection should be good clinical care, but to advance beyond encouraging vigilance and clinical acumen, investigations must uncover better indicators, links between these and sepsis, or find clinical signals for sepsis itself. Although a gold-standard signal is a laudable goal, it is most likely that science will continue to work through proxies.

#### What is known

The current identification paradigm for sepsis (Sepsis-3) makes use of established criteria (SOFA) [54]. This approach leverages a scoring system in the context of infection to provide a way to identify a population likely to benefit from therapies. This process is pragmatic but flawed. The SOFA score is twenty five years old and based on values and therapies that are in some cases outdated and are not themselves ideal criteria for organ or cellular dysfunction. There are many causes of hypoxia, altered mental status, elevated bilirubin or thrombocytopenia, each with different implications for a patient that might have sepsis. Sepsis-3 criteria are validated to mortality and ICU length of stay. However, neither mortality nor length of stay fully correlate to sepsis. Each is influenced by many other factors.

There are three essential elements to the concept of sepsis: infection, a dysregulated host response, and organ (cellular) dysfunction. Defining infection might be the least problematic, but it is imperfect. Defining a dysregulated host response implies a sound understanding of a regulated host response, but the understanding is incomplete. A stress response to injury has multiple descriptions [141, 142]. It is clear that the integrated and coordinated interactions of immune, [143, 144] neurologic, [145, 146] and endocrine systems [147] produce a series of physiologic adaptations [148]. Scoring systems attempt to describe organ dysfunctions. Although imperfect, they can provide a global index of dysfunction. Injury markers might improve this distinction [149].

Use of proxies leads to a compounding problem. As one proxy for organ dysfunction is combined with others, and then attributed as a part of a dysregulated host response resulting from infection, inaccuracies of each proxy multiply, such that the model’s performance is degraded even if each component by itself is reasonably accurate. Linking proxies and outcomes, one sees a related problem. Assume a marker picks up 90% of patients with sepsis. We want to correlate this marker with an outcome like ICU length of stay. If 90% of patients with sepsis have an increased length of stay, only 81% of those selected by the marker will have the outcome we are using to validate our model.

Sepsis does not occur in a clinical vacuum, and patient comorbidities, susceptibilities and responses (regulated and dysregulated) are expected to vary. A neutropenic or immunosuppressed patient will have a different response to bacteremia than someone with “normal” immune function. It may not be one disorder. Data suggest that there may be different phenotypes or endotypes for sepsis [75].

Outcomes validity for any scoring model is sensitive to the outcomes chosen. Sepsis-3 criteria use mortality and intensive care unit (ICU) stay of 3 days or longer [54], and many subsequent investigations have used similar outcomes to validate scoring systems. The mortality outcome presents a problem, as the goal is to identify patients that can be treated for sepsis. Improved performance of a model against mortality risks making the model a better mortality predictor or a triage tool. If a scoring system predicts mortality with 100% accuracy, it risks being irrelevant to clinicians hoping to improve survival.

*Key points:*

- Sepsis-3 criteria leverage a scoring system to identify patients likely to benefit from sepsis therapies.
- Most clinical information guiding the diagnosis of sepsis depends on proxies that may be markers rather than drivers of the dysregulated host response.
- Sepsis-3 criteria are validated against outcomes that may not be directly related to sepsis.

#### What is not known/gaps in knowledge

Available data inconclusively describe the relative beneficial and harmful effects of biologic perturbations, blurring the definitions of regulated and dysregulated. Clinical information consists of inflammatory biomarkers, endocrine abnormalities, altered metabolic signals and evidence of organ dysfunction, but the significance of each of these findings is unclear. Constructing a formulation of sepsis based on proxies doesn’t necessarily specify the direction of the abnormality. For example, elevated stimulating hormones might reflect central overstimulation or peripheral underactivity, requiring different treatments. Clinicians and scientists struggle to separate dysfunctions driving a dysregulated host response from those caused by the host response. Available scoring systems lack organ-specificity. Improvements from the addition of data need further validation.

We still know very little about which patterns in clinical information can identify patients requiring specific care to improve outcomes. Gaps include separating drivers from markers, injury from dysfunction, identifying infection and finding better characteristics for regulated and dysregulated host responses. We lack sufficient grasp of what should be important outcomes. Clinical research depends on uncovering patterns that identify patients at risk for clinically relevant outcomes and for whom interventions can change clinical trajectories. Available markers approximate interactions at a cellular and intracellular level. These proxies have yet to be mapped in a way that enables characterization of the whole organism response. We lack sufficient insight into model performance in both clinical construct validity (does the formulation approximate sepsis?) and outcome validity (does the formation accurately predict clinically meaningful outcomes?).

Patterns in response may be helpful ways to improve models and link them to valid outcomes, but key elements of these patterns are unexplained. In the example of the direction problem, sampling a panel of hormone function (e.g., a thyroid panel), rather than a single value (e.g., a thyroid stimulating hormone level), helps specifiy what the abnormality means. This is not as clear with sepsis. Recognizing patterns of clinical improvement or deterioration validates clinical suspicions. This approach only works if mechanisms are understood, an ongoing limitation. Efforts to quantify intensity [150] or clinical trajectory [151] add information to single-measurement proxies. Host response can vary in sepsis, and this variability provides useful staging information (152) and may help describe host response phenotypes (e.g., acute versus chronic critical illness) that shape clinical scoring tools and link them to outcomes, but these approaches are still speculative, requiring validation.

#### Future directions

Investigations into isolated organ dysfunctions would inform characterizations that could then be assessed in terms of causality, providing much needed insight into the mechanisms of sepsis. A focus on cellular dysfunction rather than nonspecific markers may help validate what relevant criteria establish a diagnosis whose treatment would be expected to improve outcomes. Further inquiry should explore clinical outcomes that approximate what a clinician would hope to improve by treating sepsis, such as by reversing organ dysfunctions (when they can be measured) or their effects.

Investigating the long-term effects and trajectories of organ dysfunctions could inform discussions about their nature and meaning. Interactions and more subtle data patterns (e.g., heart rate variability, pairing autonomic, endocrine and immune signals) can be investigated and may provide insight about integrated host responses to infection. How patient factors influence clinical data outcomes prediction deserves further study. The interactions of neurologc, endocrine and immune systems with critical functions such as metabolism, coagulation, and cell death, need characterization. Investigations should aim to build mechanistic models that help explain how complementary clinical data relate to each other and describe the benefits and harms of adaptation patterns. Finally, future investigations should address clinical criteria construct validitiy and outcome validity.

*Important research questions:*

- How can markers of a dysregulated host response be distinguished from drivers of that response?
- How do cellular-level processes interact to produce organism-level responses?
- What is the best way to characterize a dysregulated response to infection, optimizing clinical criteria, construct validity and outcomes validity?

## Conclusions/summary

The six questions addressed by this paper identify important issues about how to define, categorize, assess, predict, diagnose and treat sepsis. Subcommittee members, representing a broad group of clinicians from different parts of the world, addressed these considerations from a variety of perspectives. However, although the process used the common Delphi standard, it is based on the consensus of experts and is therefore subjective and prone to bias.

The accounts of what is known, the identified knowledge gaps, and the future directions for research serve as an important and prioritized resource for future sepsis investigations. Addressing these key gaps will lead to better understanding of disease epidemiology, pathobiology and individual patient heterogeneity, which will facilitate timely and accurate sepsis patient identification to deliver effective therapies, building on the progress made by the Sepsis-3 Task Force and the Surviving Sepsis Campaign.

## Data Availability

Supporting data are cited in this narrative review. Mark E. Nunnally: no competing interests. Ricard Ferrer: Dr. Ferrer is a paid Board member for Merck Sharp and Dohme, and Shionogi, has received a grant from Becton, Dickinson and Company, has received payment for development of educational presentations from Pfizer, Grifols, Thermo and Baxter, and owns stock or stock options with Grifols. Greg S. Martin: no competing interests. Ignacio Martin-Loeches: Dr. Martin-Loeches serves on advisory boards with Gilead, Merck, Pfizer and Accelerate Diagnostics. Flavia R. Machado: no competing interests. Daniel De Backer: Dr. DeBacker is past president of the European Society of Intensive Care Medicine and has received consulting fees from Fresenius Kabi. Craig M. Coopersmith: no competing interests. Clifford S. Deutschman: Dr. Deutschman reports grants from the Nationa Institute of General Medical Sciences, during the preparation of the manuscript; stock options from Enlivex, study drug supply from La Jolla Pharmaceuticals, and personal fees from Society of Critical Care Medicine, Pfizer and Sage Therapeutics, outside the submitted work.
